# Elevated MiR-222-3p Promotes Proliferation and Invasion of Endometrial Carcinoma via Targeting ERα

**DOI:** 10.1371/journal.pone.0087563

**Published:** 2014-01-31

**Authors:** Binya Liu, Qi Che, Haifeng Qiu, Wei Bao, Xiaoyue Chen, Wen Lu, Bilan Li, Xiaoping Wan

**Affiliations:** 1 Department of Obstetrics and Gynecology, International Peace Maternity & Child Health Hospital Affiliated to Shanghai Jiao Tong University School of Medicine, Shanghai, China; 2 Department of Obstetrics and Gynecology, Shanghai First People's Hospital Affiliated to Shanghai Jiao Tong University School of Medicine, Shanghai, China; University of Texas Health Science Center, United States of America

## Abstract

MicroRNAs play key roles in tumor proliferation and invasion. Here we show distinct expression of miR-222-3p between ERα-positive and ERα-negative endometrial carcinoma (EC) cell lines and primary tumors, and investigation of its relationship with ERα and other clinical parameters. In vitro, the function of miR-222-3p was examined in RL95-2 and AN3CA cell lines. MiR-222-3p expression was negatively correlated with ERα. Over-expressed miR-222-3p in RL95-2 cells promoted cell proliferation, enhanced invasiveness and induced a G1 to S phase shift in cell cycle. Furthermore, the miR-222-3p inhibitor decreased the activity of AN3CA cells to proliferate and invade. In vivo, down-regulated miR-222-3p of AN3CA cells inhibited EC tumor growth in a mouse xenograft model. Additionally, miR-222-3p increased raloxifene resistance through suppressing ERα expression in EC cells. In conclusion, miR-222-3p plays a significant role in the regulation of ERα expression and could be potential targets for restoring ERα expression and responding to antiestrogen therapy in a subset of ECs.

## Introduction

EC is the most common malignancy of the female genital system, with estimated 8,190 deaths in the USA in 2013 [1]. EC is often associated with excessive estrogen exposure, and often co-exist with, or is preceded by endometrial hyperplasia [2]. In China, the incidence of EC has been increasing with a shift to younger population due to multiple factors, such as obesity and lifestyle changes [3], [4]. Upon initial diagnosis, as 28% of the patients already have regional or distant metastasis [5]. However, the etiology of EC remains unclear. Current treatments for EC (including surgery, chemotherapy, and radiation therapy) always produce significant side effects. To date, our knowledge of this disease is still quite limited. Therefore, further elucidation of the molecular mechanisms during endometrial cancer is urgently required.

Estrogen is a classical etiological factor for EC and the pro-oncogenic effect of estrogen is mediated primarily by estrogen receptor alpha (ERα) activation of target genes that promote cell proliferation or decrease apoptosis [6]. ERα is an important marker for prognosis and is predictive of response to endocrine therapy in patients with EC [7]. The most common basis for determining risk of recurrent disease is the categorization into type I and II endometrial carcinoma [8]. Type I tumors are most frequent, characterized by endometrioid histology, low histologic grade, low Federation Internationale des Gynaecologistes et Obstetristes (FIGO) stage, and favorable prognosis. In contrast, type II tumors are characterized by nonendometrioid histology, high histologic grade and stage, and a tendency to recur, even when treated at early stage [9]. The molecular basis for type I and II cancers is only partially understood. Hyperestrogenic risk factors and positivity for ERα are common in type I in contrast to type II cancers, and ERα status is reported to be a prognostic marker in endometrial cancer [10], [11]. Decreased or absent expression of ERα is associated with extensive invasion, disease progression and poor prognosis [12]. Understanding the basis for ERα discrepancies between EC and normal endometrium will hopefully improve the understanding of endometrial carcinogenesis and facilitate research and development of novel therapy.

MicroRNAs (miRNAs) are small noncoding RNAs that silence of their cognate target genes by either degrading mRNAs or inhibiting their translation [13]. As such, miRNAs are implicated in the regulation of a variety of cellular processes, including stemness and metastasis; also, miRNAs could function as either oncogenes or tumor suppressors [14]–[16]. MiR-222-3p overexpression facilitate the growth, metastasis and invasion of a variety of malignant tumors, including breast cancer [17], [18], lung cancer [19], colorectal carcinoma [20], and melanoma [21] via genetic or epigenetic mechanisms.

In our previous study using microRNAs microarray, we found that miR-222-3p significant overexpression in ERα-negative EC cells (vs. ERα-positive EC cells). Here, we represent a comprehensive analysis of miR-222-3p expression in atypical hyperplasia, clinical EC tumor samples and normal endometrium. We investigated the regulatory effects of miR-222-3p on ERα, and explored the potential therapeutic value of miR-222-3p in EC cell lines through functional analysis. These findings provided new insights into the invasive mechanisms in EC, and encouraged exploring miR-222-3p as a target for intervention.

## Materials and Methods

### Ethics statement

The Human Investigation Ethical Committee of International Peace Maternity & Child Hospital Affiliated Shanghai Jiao Tong University approved this study. All samples were obtained from patients who signed informed consent approving the use of their tissues for research purposes after operation. Our animal research carried out was in accordance with the recommendations in the Guideline for the Care and Use of Laboratory Animals of China. The protocol was approved by the Committee on the Ethics of Animal Experiments of the Obstetrical and Gynecological Hospital affiliated Fu Dan University (Permit Number: SYXK (hu) 2008–0064). All efforts were made to minimize animal suffering.

### Cell culture

Human EC cell lines (RL95-2, AN3CA, KLE), human breast cancer cell lines MCF-7 and human embryonic kidney 293T cells were purchased from American Type Culture Collection (ATCC, Manassas, VA, USA). MCF-7 cell lines were grown in Dulbecco's modified Eagle medium (Sigma; St. Louis, MO, USA) containing 10% heat-inactivated fetal bovine serum (FBS) and 1% penicillin/streptomycin (Gibco; Auckland, NZ). All other cell lines (RL95-2, AN3CA, KLE and 293T) were cultured in Dulbecco's Modified Eagle Medium: Nutrient Mixture F-12 (DMEM/F12) supplemented with 10% FBS and 1% penicillin/streptomycin. Working cultures were maintained at 37°C in a humidified incubator with 5% CO_2_.

### Tissue collection

EC samples (n = 75) were obtained from patients who underwent surgical therapy at the International Peace Maternity and Child Health Hospital of the China Welfare Institute, affiliated to Shanghai Jiao Tong University School of Medicine, from February 2009 to March 2012. Tumor staging and histological grading were carried out using the FIGO criteria (2009) [22]. The local ethics committee approved the research project. Informed consent for the experimental use of the surgically removed samples was obtained from all patients. None of the patients received hormone therapy, radiotherapy, or chemotherapy prior to the sample collection. Following excision, tissue samples were immediately snap-frozen in liquid nitrogen and stored at −80°C until RNA extraction. Clinical and pathological data are presented in Table 1.

**Table 1:** Characteristics of the endometrial carcinoma (EC) samples.

Clinicopathologocal variables	Number of patients (%)	
	N	%
*Total*	75	100
*Age (years)*		
≥55	47	63
<55	28	37
*FIGO stage*		
Stage I	47	63
Stage II	16	21
Stage III	12	16
*Grade*		
G1	36	48
G2	26	35
G3	13	17
*Myometrial invasion*		
**<1/2**	61	81
**≥1/2**	14	19
*Nodal metastasis*		
Positive	11	17
Negative	64	83
*Estrogen receptor status*		
Positive	57	76
Negative	18	24

### Immunohistochemistry

Briefly, after deparaffinization and dehydration, specimens were boiled in 10 mM sodium citrate buffer to unmask antigens. Specimens were then blocked and incubated with primary antibody overnight at 4°C. Antibody binding was detected using Envision reagents (Boster bioengineering, Wuhan, PR China) according to the manufacturer's instructions. Primary antibodies used in this study were rabbit polyclonal anti-Ki67 (Epitomics, Burlingame, CA, USA, 1∶100 dilution), rabbit monoclonal anti-ERα (Epitomics, 1∶100 dilution), rabbit monoclonal anti-PTEN (Epitomics, 1∶100 dilution) and rabbit polyclonal anti-TIMP3 (Proteintech Group Inc., Chicago, IL, USA, 1∶50 dilution).

### RNA extraction and quantitative reverse transcription-polymerase chain reaction (qRT-PCR) for miRNA and mRNA

Total RNA was extracted from cultured cells and tissues using TRIzol® RNA Isolation Reagents (Invitrogen, Carlsbad, CA, USA). The expression of miR-222-3p was quantified by qRT-PCR using TaqMan microRNA assays (Applied Biosystems, Carlsbad, CA, USA) and normalized to endogenous control U6B. All the reagents were purchased from Applied Biosystems (Foster City, CA, USA). Mature miRNA was reverse-transcribed from total RNA using specific miRNA RT-primers in TaqMan MicroRNA Assays with reagents in the TaqMan MicroRNA Reverse Transcription kit. qRT-PCR was performed using TaqMan MicroRNA Assay primers with the TaqMan Universal PCR Master Mix and analyzed with an ABI Prism 7000 Sequence Detection System (Applied Biosystems).The cDNA was generated using Prime Script RT Reagent Kit (TaKaRa; Dalian, PRC). For quantification of ERα, pS2, PR, cyclin D1, qRT-PCR was carried out on ABI Prism 7000 Sequence Detection System (Applied Biosystems) with SYBR Premix Ex Taq (TaKaRa; Dalian, PRC). The primer sequences are listed in Table 2. For all the qRT-PCR experiments, values on the y-axis equal to 2^−ΔCt^, where ΔCt is the difference between gene Ct and normalizer gene Ct. Ct represents the threshold cycle at which fluorescence rises statistically significantly above the baseline. Gene expression data were obtained in triplicate in three independent experiments.

**Table 2:** Primers sequences for real-time PCR analysis.

mRNA	Size (bp)	Primer sequence
ERα	196	Forward5′-TTATGGAGTCTGGTCCTGTGAG-3′
		Reverse5′-TCCTCTTCGGTCTTTTCGTATC-3′
GAPDH	532	Forward5′-AGGTCGGAGTCAACGGATTTG-3′
		Reverse5′-GTGATGGCATGGACTGTGGT-3′
pS2	183	Forward5′-CATCGACGTCCCTCCAGAAGAG-3′
		Reverse5′-CTCTGGGACTAATCACCGTGCTG-3′
CyclinD1	191	Forward5′- AGGAACAGAAGTGCGAGGAGG-3′
		Reverse5′- GATGGAGTTGTCGGTGTAGATG-3′
PR	162	Forward5′-TCTACCCGCCCTATCTCAACTA-3′
		Reverse5′-AGAAGACCTTACAGCTCCCACA-3′

### Transfection

RL95-2 and AN3CA cells were washed with PBS and re-seeded in antibiotic-free growth medium for 24 h before transfection. 1×10^6^ of RL95-2 cells were transiently transfected with 100-nM miR-222-3p mimics (miR-222m) or mimics negative control (miR-222m NC) using the Lipfectamine2000 transfection reagent (Invitrogen, Carlsbad, CA, USA). 100-nM miR-222-3p inhibitor (miR-222i) or inhibitor negative control (miR-222i NC) were transfected into 1×10^6^ of AN3CA cells. SiRNA oligonucleotide duplexes were synthesized by Genephama Biotech (Shanghai, China). After 8 h, medium was replaced by normal 10% FBS DMEM/F12 medium. The oligonucleotides were as follows: has-miR-222-3p mimics (miR-222m): 5′-AGCYACAUCUGGCUACUGGGU-3′ (sense) and 5′-CCAGUAGCCAGAUGUAGCUUU-3′ (antisense); and mimics negative control (miR-222m NC): 5′-UUCUCCGAACGUGUCACGUTT-3′ (sense) and 5′-ACGUGACACGUUCGGAGAATT-3′ (antisense); and has-miR-222-3p inhibitor (miR-222i): 5′-ACCCAGUAGCCAGAUGUAGCU-3′; and inhibitor negative control (miR-222i NC): 5′-CAGUACUUUUGUGUAGUACAA-3′.

### Immunoblot analysis

For each of three independent experiments, 60-µg total protein extract was separated on 10% SDS-PAGE gels and transferred to PVDF membrane. The levels of ERα expression were evaluated by using a rabbit monoclonal anti-ERα antibody (Epitomics, 1∶2000 dilution). As a loading control, β-actin expression levels were measured using mouse monoclonal anti-actin antibody (Proteintech Group Inc., 1∶2000 dilution). The secondary horseradish peroxidase-conjugated antibodies (Santa Cruz, goat anti-rabbit, 1∶1000 dilution; Proteintech Group Inc., goat anti-mouse, 1∶4000 dilution) were detected using ECL Plus Western blotting detection reagents (Amersham Biosciences). Bands were quantified with ImageJ 1.34 software.

For evaluation of ERα expression, staining intensity was scored as 0 (negative, −), 1 (weak, +), 2 (medium, ++), or 3 (strong, +++). The extent of staining was scored as 0 (0%), 1 (1%–25%), 2 (26%–50%), 3 (51%–75%), or 4 (76%–100%), according to the percentage of the positively stained areas in relation to the whole tumor area. Two pathologists who were blinded to details regarding patient background assessed the results.

### Proliferation assay

RL95-2 and AN3CA cells were seeded into a 96-well plate (5×10^3^/well). After 24 h incubation, RL95-2 and AN3CA cells were transfected with miR-222m or miR-222i duplexes using the Lipfectamine2000 transfection reagent (Invitrogen) respectively. At 24 h post-transfection, both the cells were treated with raloxifene (10–100 µM) or phenol red-free medium containing 5% charcoal-stripped FBS (HyClone Laboratories; Logan, Utah, USA) and 0.1% alcohol as a control for 4 days. Then cells were subjected to 3-(4,5-dimethylthiazol-2-yl)-2,5-diphenyltetrazolium bromide (MTT) (Sigma). Absorbance was examined at 490 nm using a SpectraMax 190 microplate reader (Molecular devices, Sunnyvale, CA, USA). Each individual experiment was repeated three times in triplicate.

### Cell cycle analysis

1×10^6^ of RL95-2 and AN3CA cells were fixed with 70% ethanol at 72 h after transfection and stained with 25 µg/mL propidium iodide (Roche Molecular Biochemicals, Indianapolis, IN) in fluorescence-activated cell sorting buffer (phosphate-buffered saline containing 0.1% bovine serum albumin, 0.05% Triton X-100 and 50 µg/mL RNase A). Both the cells were analyzed using FACSCalibur and Cell Quest Pro Software (BD Biosciences, San Jose, CA). Experiments were performed in triplicate in three independent experiments.

### Cell invasion assay

Cell invasion was evaluated using transwell chamber assay (Millipore, Billerica, MA, USA) according to the manufacturer's instruction. For invasion assay, totally 5×10^4^ of RL95-2 and AN3CA cells were seeded on an 8-µm pore size transwell insert coated with extracellular matrix (ECM) (1∶6) (BD Biosciences). After incubated at 37°C for 48 h, the cells adherent to the upper surface of the filter were removed using a cotton applicator, then stained with crystal violet, and the values obtained were calculated by averaging the total numbers of cells from triplicate determinations.

### Colony formation assay

For the plate colony formation assay, 200 cells/well of RL95-2 and AN3CA trypsinized cells were seeded into 6-well plates and incubated in a humidified 5% CO_2_ atmosphere for 2 weeks. Then, cells were rinsed with PBS and fixed with 1% formaldehyde for 30 min at room temperature. Fixed cells were stained with 0.5% crystal violet. Colonies (containing ≥50 cells; ≥3 mm^2^) were counted. Experiments were performed in three independent experiments.

### Enzyme-linked immunosorbent assay (ELISA) for determination of matrix metalloproteinase-2 (MMP-2) and matrix metalloproteinase-9 (MMP-9)

Non-transfected and transfected RL95-2 or AN3CA cells were seeded into 24-well plates at a density of 1×10^6^ cells/ml, and cultured as previously described. MMP-2 and MMP-9 in the cell culture medium were examined using the Quantikine Human MMP-2 Immunoassay (R&D Systems, Minneapolis, MN) and the human MMP-9 ELISA Kit (Bender Med System Co., CA, USA), respectively. Values were read using microplate reader (Bio-Rad model 680, USA). Each experiment was repeated three times in triplicate.

### Construction of reporter plasmids and luciferase assays

The 3′ UTR of the human ERα gene was PCR amplified using the following primers: 5′-TCAGAG-cctattgttggatattgaatgacagacaatcttatgtagcaaagattatgcctgaaaagggatcc-GC-3′ (forward) and 3′-GGCCGC-ggatcccttttcaggcataatctttgctacataagattgtctgtcattcaatatccaacaatagg-C-5′ (reverse) and cloned between the Xho1 and Not1 sites of the psiCHECK 2 Vector (Promega, Madison, WI, USA), giving rise to the p3′UTR-ERα plasmid. The construct was used to generate, by inverse PCR, the p3′UTRmut-ERα plasmid (primers: forward: 5′-TCGAG-cctattgttggatattgaatgacagacaatctttacatcgaaagattatgcctgaaaagggatcc-GC-3′ and reverse: 3′-GGCCGC-ggatcccttttcaggcataatctttcgatgtaaagattgtctgtcattcaatatccaacaatagg-C-5′).

RL95-2 cells were co-transfected with 200 ng of p3′UTR-ERα plasmid and with p3′UTRmut-ERα plasmid and 200 ng of Renilla luciferase plasmid by using Lipofectamine 2000 (Invitrogen). Cells were harvested 48 h post-transfection and assayed with Dual Luciferase Assay (Promega) according to the manufacturer's instructions. Three independent experiments were performed in triplicate.

### Tumorigenicity assays in nude mice

The Ethic Committee for Animal Experimentation of Shanghai Jiaotong University approved all experimental protocols. A total of 5×10^6^ cells suspended in 100 µl 1× PBS were injected into subcutaneously to the interscapular area in 4-week old female BALB/C athymic nude mice. A group of mice (n = 4) received AN3CA cells (1×10^7^) transfected with LV-has-miR-222-3p-down (miR-222-3p inhibitor in lentivirus vector; LV-miR-222i). Another negative control group received AN3CA cells (1×10^7^) transfected with LV-has-miR-222-3p-down NC (lentivirus vector alone; LV-miR-222i NC). The size of tumors was measured weekly for 4 weeks. Mice were sacrificed at 30 days post-injection. Tumors were excised and measured. Tumor volume (cm^3^) was calculated by using the following formula: (the longest diameter)×(the shortest diameter)^2^×0.5.

### Statistical analysis

All data are expressed as mean ± standard deviation (SD). Statistical evaluation of the data was performed with one-way ANOVA. Pair-wise comparisons were conducted by Student's t test. The P value less than 0.05 were considered statistically significant. All analyses in the study were evaluated with SPSS version 17.0 software (Chicago, IL, USA).

## Results

### Expression of miR-222-3p is up-regulated in EC

**Figure 1:**
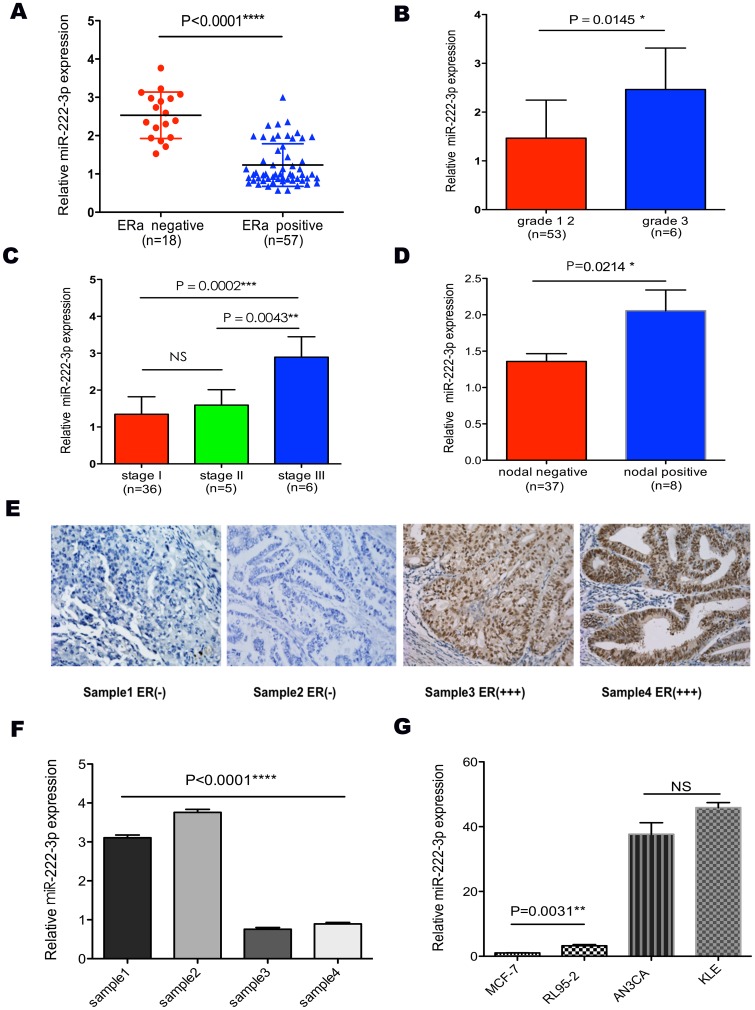
The expression of miR-222-3p correlated with ERα and clinicopathological parameters in endometrial carcinoma. (A) MiR-222-3p expression level was much higher in ERα-negative ECs than in ERα-positive samples. (B, C and D) Expression of miR-222-3p across different grades, stages and nodal metastasis status. MiR-222-3p expression level was positively correlated with poor clinicopathological parameters. (E and F) MiR-222-3p and ERα expression were inversely related in ECs and normal endometrium tissues. These tissues were analyzed for miR-222-3p expression by TaqMan based qRT-PCR, followed by immunohistochemistry for ERα as described in “Materials and Methods”. (G) Expression of miR-222-3p in cells with different ERα status. MCF-7 and RL95-2 cells were ERα-positive, while KLE and AN3CA cells were ERα-negative. Conversely, miR-222-3p expression was negative-related with ERα status. Bars are standard deviation (SD). The experiments were repeated three times. ** P<0.01 vs. RL95-2, MCF-7. ****P<0.0001 vs. RL95-2, KLE, AN3CA. * P<0.05, ** P<0.01, *** P<0.001, **** P<0.0001.

Our results showed that miR-222-3p expression was much lower in ERα-positive than in ERα-negative EC tissue samples (P<0.0001, Fig. 1A), and level of miR-222-3p expression was correlated inversely with ERα expression (Fig. 1A). Since miR-222-3p was higher in ERα negative ECs than in ERα positive cases, we further studied miR-222-3p expression level and its association with clinicopathologic parameters in ECs. The level of miR-222-3p expression was lower in tumors of lower grades (1 and 2 vs. 3, P = 0.0145, Fig. 1B) and earlier stage (I vs. II, P>0.05; II vs. III, P = 0.0043; I vs. III, P = 0.0002; Fig. 1C). In addition, miR-222-3p was associated positively with lymphatic nodal metastasis (P = 0.0214, Fig. 1D).

There was a significant rising of miR-222-3p expression in ERα-negative EC cells (vs. ERα-positive EC cells; P<0.0001). As shown in Fig. 1G, the expression of miR-222-3p in AN3CA cells were 12-fold (P<0.0001) and 38-fold (P<0.0001) than that of RL95-2 and MCF-7 cells, respectively; the expression of miR-222-3p in KLE cells was 14-fold (P<0.0001) and 46-fold (P<0.0001) than that of RL95-2 and MCF-7 cells, respectively; the expression of miR-222-3p in RL95-2 cells was 3-fold (P = 0.0031) than that of MCF-7 cells. These results suggest that miR-222-3p could be an oncogenic microRNA in EC.

### MiR-222-3p promoted proliferation and invasion potential in cultured EC cells

**Figure 2:**
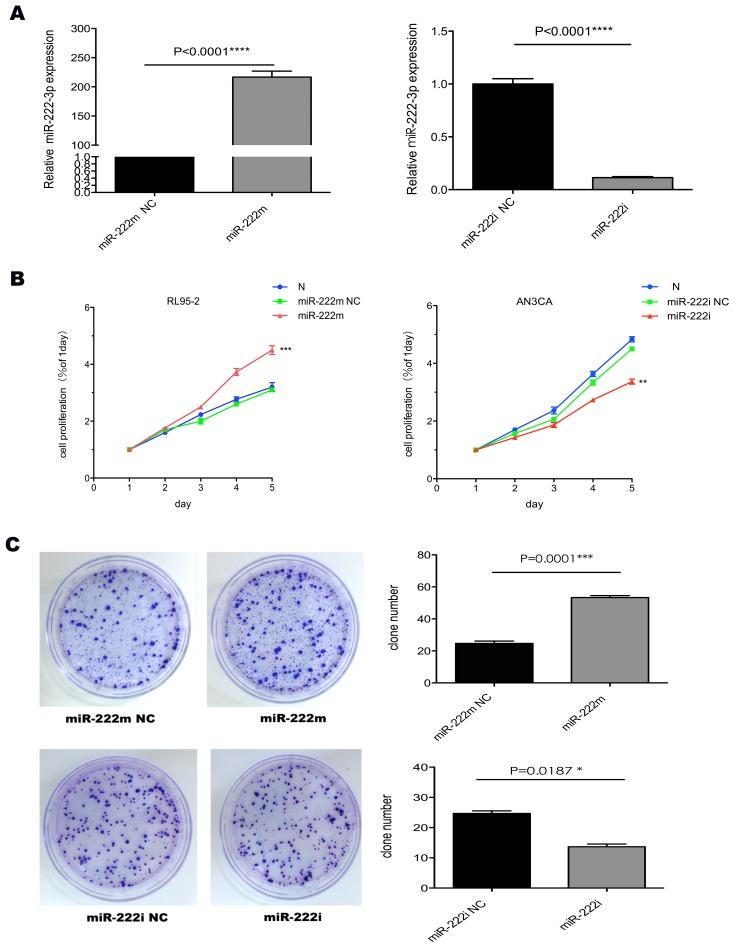
Transfection efficiency and cell proliferation after miR-222-3p expression changes. (A) qRT-PCR in RL95-2 and AN3CA cells after enforced and silenced expression of miR-222-3p for 48 h. (B) Cells with different miR-222-3p expression level were assayed for proliferation by MTT method at various time points. (C) Overexpression of miR-222-3p led to a significant increase in anchorage-independent colony forming ability of RL95-2 cells. Colony formation (≥50 cells) was assessed using a colony counter. Knockdown of miR-222-3p led to a decrease in anchorage-independent colony forming ability of AN3CA cells. * P<0.05, ** P<0.01, *** P<0.001, **** P<0.0001.

To directly demonstrate the functional role of miR-222-3p in tumorigenesis, we over-expressed or silenced miR-222-3p in RL95-2 and AN3CA cells respectively. Transfection efficiency of RL95-2 and AN3CA cells was detected at 72 h post-transfection (Fig. 2A). The proliferation assay and colony formation assay showed that overexpression of miR-222-3p in RL95-2 cells accelerated the cell growth (Fig. 2B). Oppositely, knockdown of miR-222-3p inhibited cell growth in AN3CA cells (Fig. 2C).

**Figure 3:**
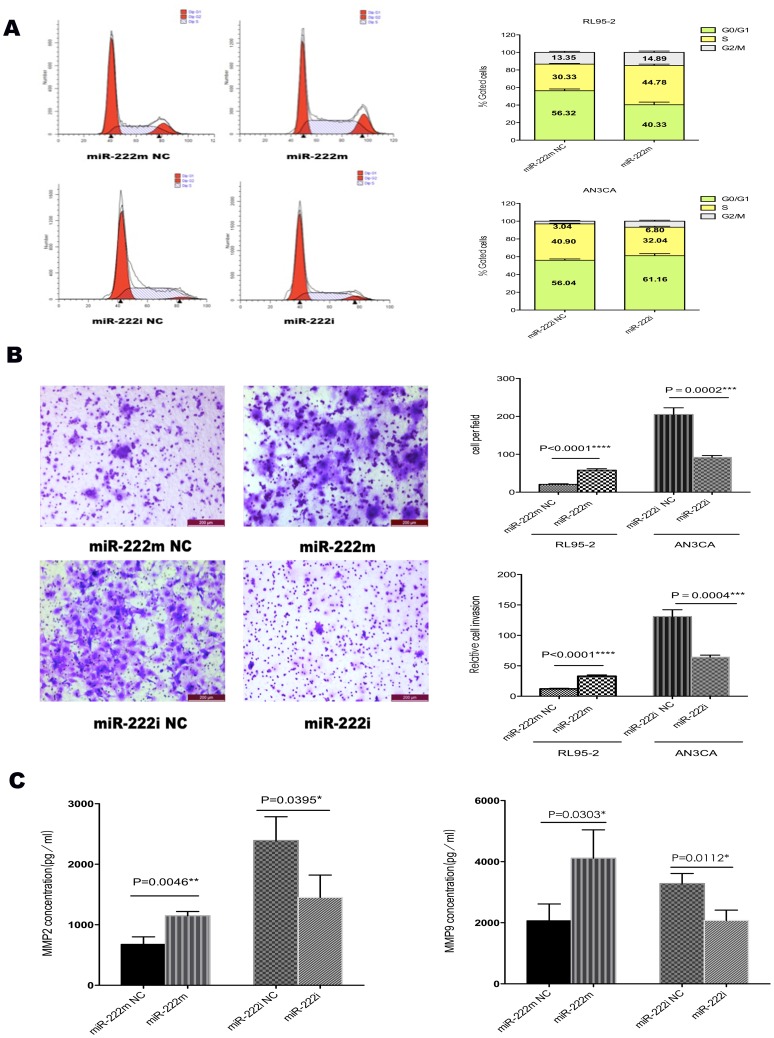
Effects of miR-222-3p on cell cycle, invasion ability and secretion of MMPs. (A) MiR-222m-transfected RL95-2 cells and miR-222i-transfected AN3CA cells were subjected to fluorescence-activated cell sorting analysis, and the relative G1, S, and G2/M compartments calculated. Data represent the average percentage of cells in each compartment in three independent experiments, each performed in triplicate. (B) Representative photos and statistical plots of transwell assays in RL95-2 cells transfected with miR-222m and miR-222m NC, and AN3CA cells transfected with miR-222i and miR-222i NC (×100). More cells traversed the transwell membrane in miR-222m transfected cells, and fewer did so in the miR-222i transfected AN3CA cell line. To exclude the effect of cell proliferation, relative cell invasion ability of RL95-2 and AN3CA cells was analyzed by normalizing relevant invasive cell numbers to 48 h MTT OD values. The results in both cells showed that high level of miR-222-3p promoted the ability of relative cell invasion. (C) MMP-2 and MMP-9 decreased significantly in cells transfected with miR-222m and increased in miR-222i transfected cells. Data are expressed as mean±SD. * P<0.05, ** P<0.01, *** P<0.001, **** P<0.0001.

Furthermore, cell cycle analyses indicated that RL95-2 cells overexpressing miR-222-3p had a significant increase in S phase population, as compared with miR-222m NC cells, with a concomitant decrease of the G1 portion (Fig. 3A). On the contrary, inhibiting miR-222-3p resulted in an accumulation of cells in G0/G1 phase and a decrease in S phase in AN3CA cells (Fig. 3A).

RL95-2 cells transfected with miR-222m had increased invasion rate compared with miR-222m NC (Fig. 3B). MiR-222i transfection in the AN3CA cell line also decreased invasive ability (Fig. 3B). Excluding the influence of cell proliferation in different treatment, relative cell invasion ability was also enhanced in RL95-2 cells with miR-222-3p overexpressing (Fig. 3B). By contrast, relative cell invasion ability weakened with miR-222i transfection in AN3CA cells (Fig. 3B). Ectopic expression of miR-222-3p impaired invasion and the secretion of MMP2 and MMP9 in EC cells (Fig. 3C). Overexpression of miR-222-3p in RL95-2 cells increased MMP-2 and MMP-9 levels (Fig. 3C). By contrast, dampening of miR-222-3p reduced the secretion of MMP-2 and MMP-9 in AN3CA cells (Fig. 3C).

### MiR-222-3p directly targets ERα and inhibits other EREs (estrogen regulated elements) genes

**Figure 4:**
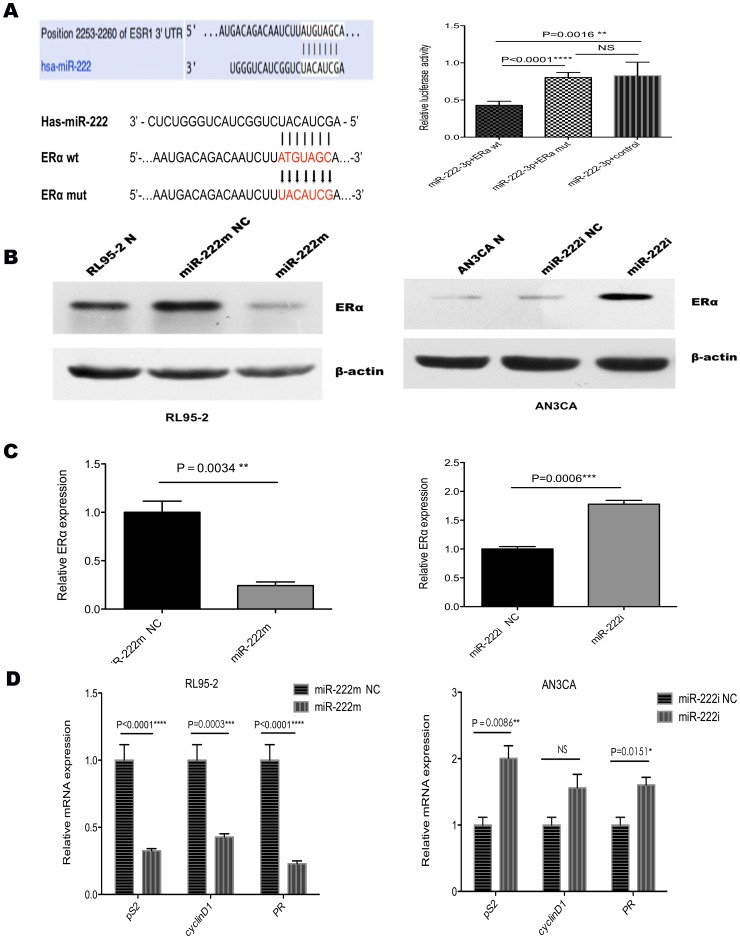
MiR-222-3p negatively regulates ERα. (A) ERα 3′ UTRs is a target of miR-222-3p. P3′UTR-ERα luciferase constructs, containing a wild type or mutated type ERα 3′ UTRs, were transfected into RL95-2 cells. Relative repression of firefly luciferase expression was standardized to a transfection control. The reporter assays were performed three times with essentially identical results. (B and C) MiR-222-3p and ERα levels were analyzed by qRT–PCR and western blot method, respectively. ERα levels decreased when miR-222-3p was upregulated in response to the miR-222m in RL95-2 cells, whereas the reverse was observed for ERα expression when miR-222-3p was knocked down in AN3CA cells. (D) The expression of pS2, cyclin D1 and PR was down regulated after miR-222m transfection in RL95-2 cell lines. Oppositely, in AN3CA cells, these ERα downstream genes were increased with miR-222-3p dampened. * P<0.05, ** P<0.01, *** P<0.001, **** P<0.0001.

A search for potential targets of miR-222-3p using miRanda, PicTar, and TargetScan revealed ERα as a target for miR-222-3p (Fig. 4A). We constructed luciferase reporters with two targeting sequences of wild type (pmiR-ERα wt; Fig. 4A) and mutant type (pmiR-ERα mut; Fig. 4A). Increased expression of miR-222-3p upon transfection, confirmed by qRT- PCR (Fig. 2A), significantly affected luciferase expression, measured as relative luciferase activity (Fig. 4A). Conversely, when we performed luciferase assays by using a plasmid harboring the 3′ UTR of ERα mRNAs, where the binding site for miR-222-3p was inactivated by site-directed mutagenesis, we observed a consistent reduction in miR-222-3p inhibitory effect in RL95-2 cells (P<0.0001, Fig. 4A).

Both qRT-PCR and western blot analysis revealed that ectopic expression of miR-222-3p in RL95-2 cells leads to a robust down-regulation of ERα (Fig. 4B and Fig. 4C), while inhibiting miR-222-3p resulted in a significant elevation in expression of ERα (Fig. 4B and Fig. 4C). Furthermore, increasing level of miR-222-3p expression, decreased effectively downstream of ERα regulating genes expression, such as pS2 (P<0.0001), cyclinD1 (P = 0.0003) and PR (P<0.0001) (Fig. 4D). In contrast, as miR-222-3p decreased in AN3CA cells, the expression of pS2 (P = 0.0086), cyclinD1 (P>0.05) and PR (P = 0.0151) were increased (Fig. 4D).

### Inhibiting miR-222-3p decreases tumor growth in a mouse xenograft model

**Figure 5:**
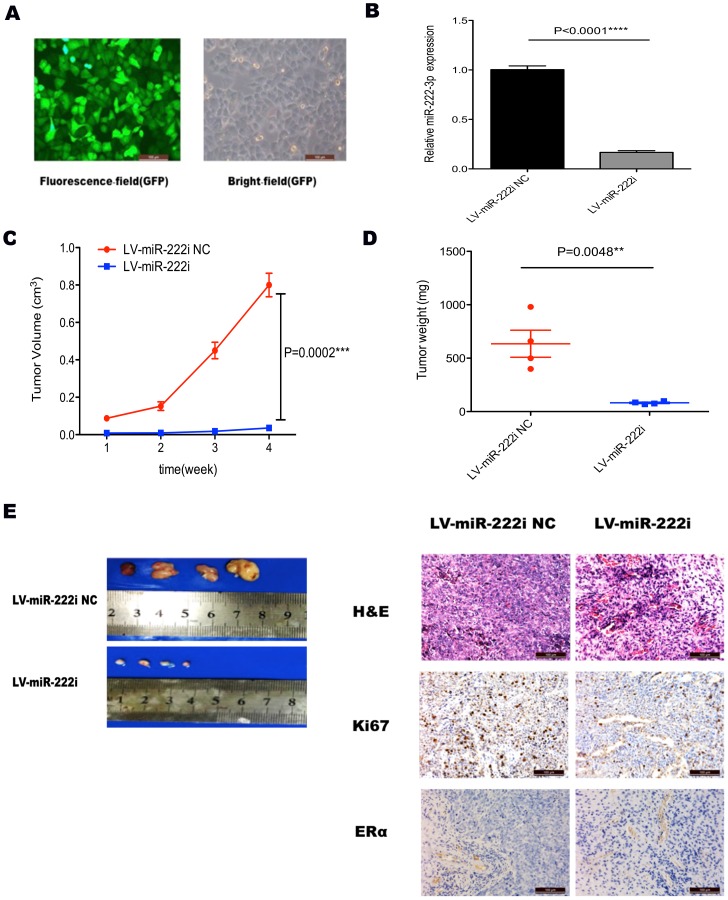
Tumorigenicity assay in nude mice. (A) Stable transfection of AN3CA cells with LV-miR-222i and LV-miR-222i NC. The percentage of transfected cells with fluorescence was >95%. (B) qRT-PCR analysis of miR-222-3p expression after transfecting with LV-miR-222i and LV-miR-222i NC in AN3CA cells. **** P<0.0001. (C) Tumor growth in nude mice. AN3CA cells with different treatment were injected subcutaneously into the interscapular area of nude mice. The short and long diameters of the tumors were measured weekly and tumor volumes (cm^3^) were calculated. *** P<0.001. (D) Weight of tumors in nude mice. **P<0.01 vs. either no transfection or control vector. (E) The nude mice with tumor formation and representative HE staining histopathologic image of tumor tissues in mice (upper panel, 200×). The expression of Ki67 and ERα in the tumor was detected by immunohistochemical techniques (200×).

To investigate the tumorigenic potential of miR-222-3p, we transfected AN3CA cells, which express high level of miR-222-3p, with LV-miR-222i (Fig. 5A). LV-miR-222i significantly decreased the expression of miR-222-3p in AN3CA cells by nearly 85% (Fig. 5B), and decreased the size and weight of the xenograft tumor as compared with LV-miR-222i NC treated group (Fig. 5C and Fig. 5D).

Furthermore, representative H&E staining was shown in Fig. 5E. The expression of Ki67, a measure for tumor cell proliferation, was decreased by LV-miR-222i in xenograft tumor (Fig. 5E). IHC results also showed that miR-222-3p knockdown lightly increased PTEN and TIMP3 expression in vivo (Fig. S1). Additionally, ERα protein expression in the xenograft was not affected by LV-miR-222i (Fig. 5E).

### MiR-222-3p affects raloxifene sensitivity in RL95-2 and AN3CA cells

**Figure 6:**
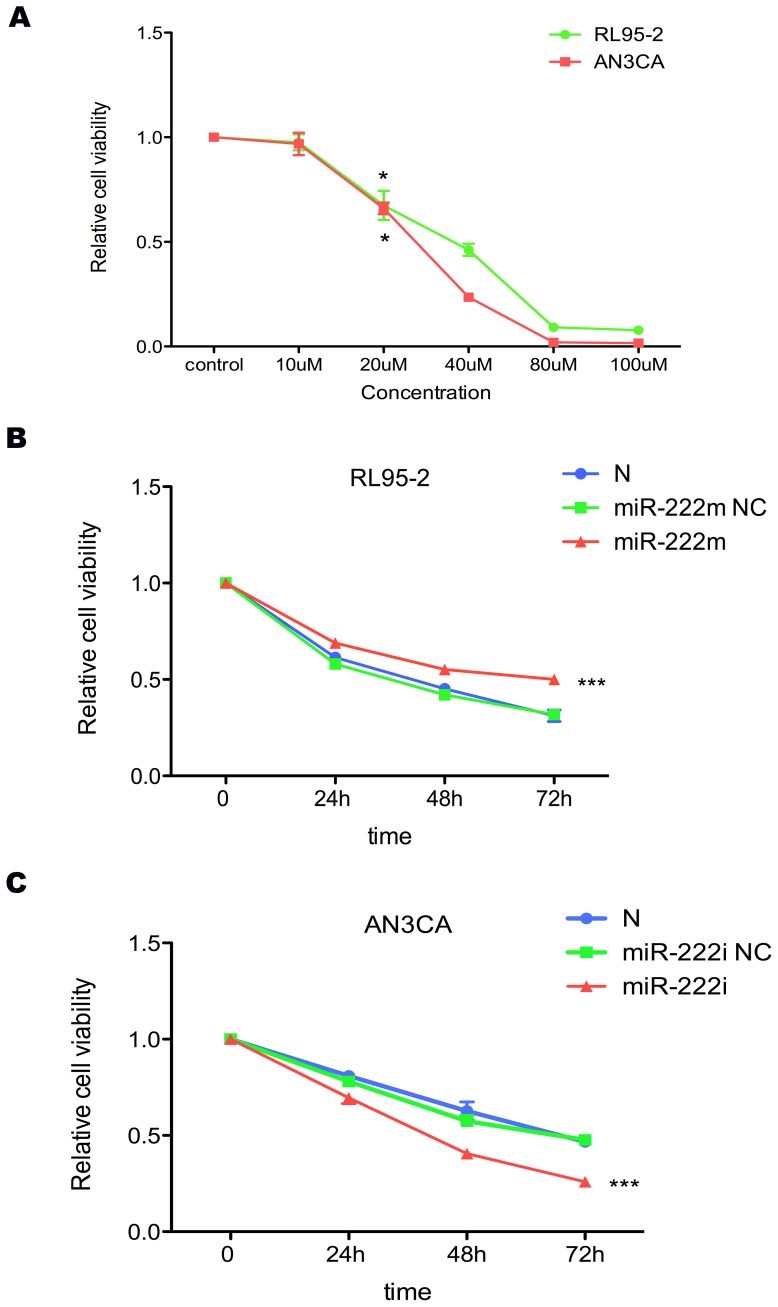
The effects of raloxifene and miR-222-3p on RL95-2 and AN3CA cells. (A) Cell viability was significantly decreased in RL95-2 and AN3CA cells treated with raloxifene. A. Raloxifene (20-µM) induced 40% inhibition of cell viability after 48 h treatment. * P<0.05. (B and C) After 24 h transfection, cells were treated with 20-µM raloxifene. At next 24 h, 48 h and 72 h, cell viability was detected by MTT assay. With miR-222-3p increasing, the cell viability of RL95-2 cells was much higher, showing less sensitivity to raloxifene. In contrast, AN3CA cells became more sensitive after miR-222-3p being inhibited. *** P<0.001.

Significant inhibition of cell growth occurred within 48 h of exposure to 20-µM raloxifene in both RL95-2 and AN3CA cells (RL95-2, P = 0.0138; AN3CA, P = 0.0162; Fig. 6A). At 48 h, 20-µM raloxifene inhibited cell growth by nearly 40%. With miR-222-3p up-regulated, RL95-2 cells showed less sensitivity to raloxifene (P = 0.0002, Fig. 6B). In contrast, AN3CA cells were more sensitive after miR-222-3p being inhibited (P = 0.0007, Fig. 6C).

## Discussion

EC remains a major cause of cancer-related morbidity and mortality among women [23]. Worldwide, it is estimated that nearly 200,000 new cases of endometrial cancer are diagnosed annually [24]. In North America and Europe, EC is the most common gynecologic malignancy [25].

Estrogen is a classic etiological factor for endometrial tumorigenesis [26]. Despite the normal and beneficial physiological actions of endogenous estrogen in women, abnormally high estrogen levels are associated with the increased incidence of certain types of cancer, especially those of the breast and endometrium [6]. The binding of estrogen to ERs induces conformational changes in protein structure that allow receptor dimerization and interaction with coactivators. The pro-oncogenic effect of estrogen is mediated primarily by ERα activation of target genes that promote cell proliferation or decrease apoptosis [6].

In EC, deregulated ERα caused by genomic or epigenetic aberrations was a prevalent phenomenon, which reduced the expression of ERα and associated with high stage and poor differentiation [11], [27]. Recent studies indicated that miRNAs are atypically expressed in virtually all cancers, including ECs [28]. Leivonen reported that five ERα-regulating miRNAs (e.g. miR-18a, miR-18b, miR-193b, miR-302c, and miR-206) directly targeted ERα in the 3′UTR [29]. Dampening of the ERα signaling by let-7 miRNAs inhibited cell proliferation and subsequently triggered the cell apoptotic process in MCF7 cells [30]. Other studies have demonstrated that miR-22 overexpression leads to a reduction of ERα level, at least in part by inducing mRNA degradation, and compromises estrogen signaling, as exemplified by its inhibitory impact on the ERα-dependent proliferation of breast cancer cells [31], [32]. In breast cancer, miR-222-3p directly repressed ERα and knockdown of miR-222-3p sensitized MDA-MB-468 cells to tamoxifen-induced cell growth arrest and apoptosis [17].

In this study, we tested 75 cases of EC samples, and demonstrated up-regulation of miR-222-3p in ERα-negative EC tissues. Also, miR-222-3p overexpression is correlated to higher grades, later stages and more nodal metastasis. Furthermore, miR-222-3p expression was significantly higher in ERα-negative cells, AN3CA and KLE, than in those of ERα-positive cells. By ectopic expressing miR-222-3p, the potential of cell proliferation and invasion was apparently enhanced in RL95-2 cells. In vivo, dampening of miR-222-3p could significantly inhibit tumor growth. Unexpectedly, we did not detect significant upregulation of ERα protein upon LV-miR-222i treatment.

In a previous report, Zhao et al. have demonstrated that ERα is suppressed by miR-221 and miR-222-3p [17]. In the current study, we found that miR-222-3p also inhibits ERα expression in EC cell lines. Unlike Zhao's study, we found that miR-222-3p inhibited ERα expression at both protein and mRNA level in EC cells. The downstream genes of ERα, including PR, cyclinD1 and pS2, were also inhibited after miR-222-3p ectopic expression in our study.

Most previous studies suggest that miR-222-3p acts as an oncogene. Elevated miR-222-3p expression has been found in highly metastatic lung cancer [19] and in glioblastoma [33]. Consistent with these published findings, our results support the concept that overexpression of miR-222-3p could increase cell proliferation, enhance invasiveness and promotes the transition from G1 to S phase. Factors previously reported to be regulated by miR-222-3p, including PTEN [19], TIMP3 [19], TRPS1 [34], CDKN1C/p57 [20] and p27kip1 [35] might also contribute to its oncogenic effect. PTEN is inactivated in certain malignant tumors, resulting in Akt hyper-activation, thereby promoting cell proliferation, inhibiting of apoptosis and enhancing cell invasion and radio resistance [36], [37]. MicroRNA, specifically miR-221 and miR-222-3p have been established as regulators of PTEN expression [19], [38].

Since RL95-2 cells enhanced invasive potential after miR-222-3p upregulated, we found that expression of MMP-2 and MMP-9 was increased. In several malignancies, MMPs have been linked to aggressive behavior, and gelatinases (MMP-2 and MMP-9) in particular are prognostic factors in EC [39], [40]. These results indicated that miR-222-3p could enhance invasive potential of ECs via promoting MMP-2 and MMP-9 secretion.

Besides the oncogenic role of miR-222-3p in vitro, tumor formation assay confirmed that decreased miR-222-3p expression could inhibit the proliferation of EC cells in a mouse xenograft model. At 1 week after injection, there were tumors in the interscapular area of mice; from 2 weeks, volume of tumors in LV-miR-222i was much smaller than LV-miR-222i NC, and in the following weeks the differences became much apparent. At 4 weeks, volume of tumors in LV-miR-222i NC was nearly 100 fold than that of LV-miR-222i. Higher proliferation in cells treatment with LV-miR-222i NC was also evident in immunohistochemical staining of Ki67. Moreover, the expression of PTEN and TIMP3 was increased in LV-miR-222i lightly. Interestingly, we did not detected apparent upregulation of ERα protein upon LV-miR-222i treatment. Given that many other mechanisms like single nucleotide polymorphism [41] and promoter hypermethylation [42] were involved, we hypothesize miR-222-3p overexpression was one of the reasons for ERα loss in AN3CA cells. Taken together, these results indicate that miR-222-3p is a crucial oncogene and may be an important determinant of ERα status in EC.

Except these routine therapies, SERMs (selective oestrogen-receptor modulators) were another choice for EC patients, which could bind the ER and modulate ER-mediated gene transcription [43]. In general, patients with ERα–positive respond favorably to SERMs; however, loss of ERα often showed SERMs resistance. As miR-222-3p directly inhibited ERα protein expression, we then further explored whether alterations of miR-222-3p have effects on cellular reaction to raloxifene (a SERM already in clinical use) in EC cells. We found that increased miR-222-3p induced resistance to raloxifene in RL95-2 cells, while down-regulation of miR-222-3p restored sensitivity of AN3CA cells to raloxifene via promoting notably cell apoptosis. Our results demonstrated that miR-222-3p overexpression was a novel mechanism for raloxifene resistance in EC patients.

In summary, our findings confirmed proto-oncogenic role of miR-222-3p. MiR-222-3p was overexpressed in ERα-negative EC tumors and was associated with high grade, late stage and nodal metastasis. Up-regulating miR-222-3p promoted cell proliferation, enhanced invasiveness and induced a G1 to S phase transition. Down-regulated miR-222-3p of AN3CA cells inhibited EC tumor growth in a mouse xenograft model. High level of miR-222-3p was a mechanism for raloxifene resistance in EC therapy. Therefore, miR-222-3p could serve as potential therapeutic targets for a subset of ERα-negative ECs and might be developed as a biomarker for EC.

## Supporting Information

Figure S1 **IHC of PTEN and TIMP3 in tumor tissues of nude mice.** (A) Alterations in PTEN and TIMP3 were confirmed by IHC (200×). After miR-222-3p being inhibited by LV-miR-222i, the expression of PTEN and TIMP3 were lightly increased in tumor tissues, as compared with LV-miR-222i NC transfected group. All experiments were repeated at least three times.
(TIF)
