# Supplementary figures and images for: Elevated MiR-222-3p Promotes Proliferation and Invasion of Endometrial Carcinoma via Targeting ERα
Source: PLoS One. 2014 Jan 31;9(1):e87563. doi: 10.1371/journal.pone.0087563 (PMC3909214; doi:10.1371/journal.pone.0087563)

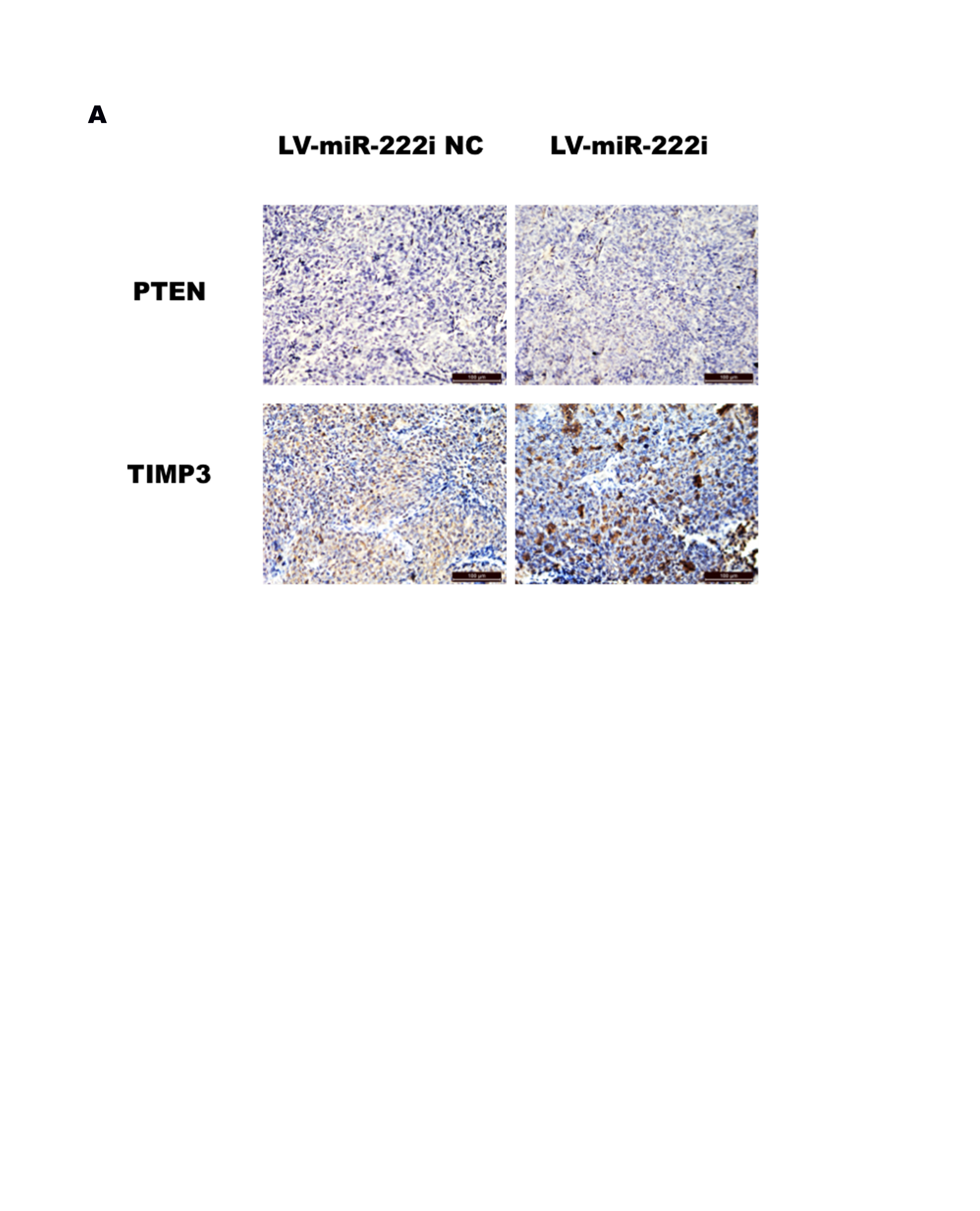

Supplement: Figure S1 — IHC of PTEN and TIMP3 in tumor tissues of nude mice. (A) Alterations in PTEN and TIMP3 were confirmed by IHC (200×). After miR-222-3p being inhibited by LV-miR-222i, the expression of PTEN and TIMP3 were lightly increased in tumor tissues, as compared with LV-miR-222i NC transfected group. All experiments were repeated at least three times. (TIF) [file pone.0087563.s001.tif]
